# Quantitation of the ROS production in plasma and radiation treatments of biotargets

**DOI:** 10.1038/s41598-019-56160-0

**Published:** 2019-12-27

**Authors:** Wan-Ook Ji, Min-Ho Lee, Gon-Ho Kim, Eun-Hee Kim

**Affiliations:** Department of Nuclear Engineering, Seoul National University, 1 Gwanak-ro, Gwanak-gu, Seoul 08826 Republic of Korea

**Keywords:** Biophysical chemistry, Biomedical engineering

## Abstract

Medical treatment utilizing non-thermal plasma is based on the production of reactive oxygen species (ROS) and their interactions with biomatters. On the basis of empirical data from practices, plasma treatment has been planned with regard to the setup of a plasma generator’s parameters, including gas combination, gas-flow rate, and applied voltage. In this study, we quantitated plasma treatment in terms of the *plasma dose* on the target matter, which can be contrasted with the *radiation dose* to targets under radiation exposure. We measured the OH radical production in cell culture medium and intracellular ROS production from plasma treatment in comparison with those from X-ray exposure. The clonogenic cell deaths from plasma and X-ray exposures were also compared. In plasma treatment, the clonogenic cell death was better predicted by intracellular ROS production rather than by medium OH production.

## Introduction

Plasma is a fully or partially ionized gas medium. In thermal plasma, heavy particles (positive ions and neutral atoms) and electrons are at the same temperature, generally reaching up to thousands of kelvins. Non-thermal plasma has the ions and neutrals at a much lower temperature (~room temperature) than electrons. In the last decade, non-thermal plasma has been utilized in medical treatments, such as microorganism deactivation, wound healing, blood coagulation, dental cavity treatment, angiogenesis suppression, and cancer treatment^1–5^. The non-thermal plasma was available from different source devices including floating-electrode dielectric barrier discharge, atmospheric pressure plasma jet (APPJ), and plasma needle^1,4^.

Non-thermal plasma affects cancer cells by generating diverse mediators including charged particles, reactive oxygen species (ROS), reactive nitrogen species (RNS), UV, and electric fields etc^1,6–10^. In medical uses, plasma is prescribed at low intensities and thus the mediators except ROS and RNS have minimal effect^8–10^. Earlier studies explored biological mechanisms behind the effectiveness of RONS in cancer treatment with plasma and found that the plasma-generated ROS mostly contributes to accumulating the oxidative stress and finally inducing cell death^11–16^. Chemical prescription of 100 times stronger NO produced by non-thermal plasma could not induce any growth inhibition of cancer cells^16^.

Non-thermal plasma and ionizing radiation operate a common pathway to cell-killing effect, that is, ROS production. Non-thermal plasma generates the OH radical in gaseous form and transfers the radicals to the medium^17–19^. The OH radical plays an important role in plasma medicine because of its higher oxygen potential and stronger disinfection power as compared with the other oxidative species^20,21^. Notably, the OH radical is a major mediator for DNA damage in cells under exposure to radiation of low linear energy transfer (or low LET), such as X-rays and γ-rays^22,23^. As a common physicochemical factor produced in plasma and radiation treatments, the OH radical production can indicate the extent of cellular exposures to plasma and radiation.

Plasma treatment is prescribed in terms of the operational setup of the source device with regard to gas combination, gas-flow rate, applied voltage, and treatment duration, which contrasts with the radiation treatment prescribed in terms of radiation dose. The parametric choices are based on the empirical results. In this study, we evaluated the effects of individual parameters, such as gas-flow rate, applied voltage, and treatment duration, of a plasma production device on the radical production and clonogenic death of *in vitro* cells. We also sought the radiation dose levels that cause the comparable radical productions and clonogenic cell deaths with the plasma exposures under different parametric combinations in operation of a plasma source device.

## Results and Discussion

### DMPO-OH production by X-ray exposure

**Figure 1:**
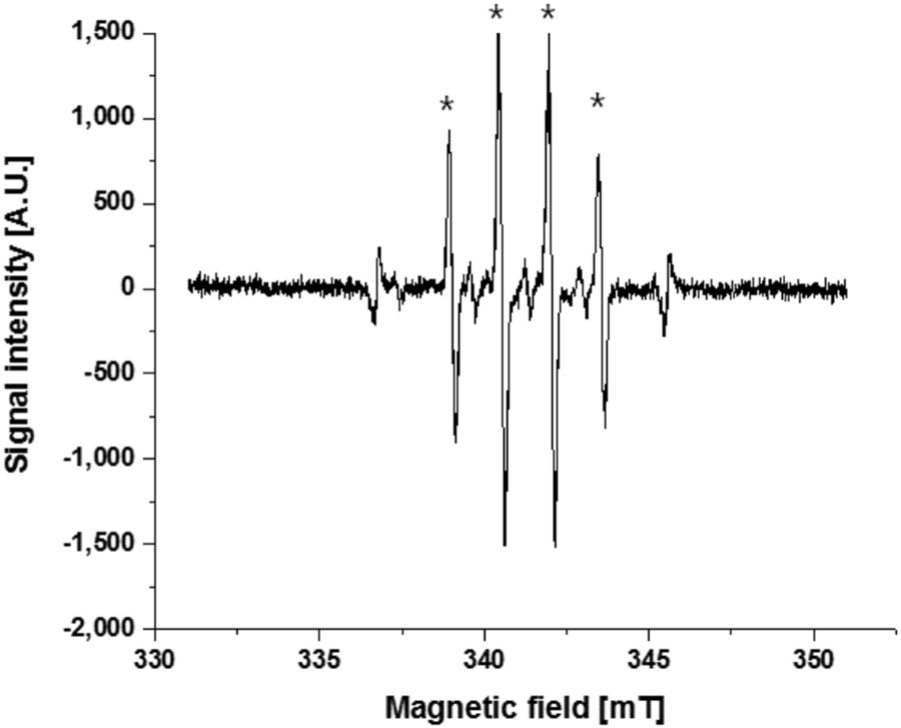
ESR measurement after exposure of the culture medium to 50 Gy of X-rays. The asterisked four peaks are the DMPO-OH signals and the outer two peaks are the Mn markers.

The production of OH radicals is difficult to detect because of their short half-life of some μs^24–27^. The OH radicals maintain a rather long half-life (870 s)^28^ when they are combined with the 5,5-dymethyl-1-pyrroline-N-oxide (DMPO). The compound DMPO-OH can be detected by electron spin resonance (ESR) spectrometry^29–32^. Figure 1 presents the DMPO-OH signals from the cell culture medium exposed to X-rays at 50 Gy. The ESR measurement was made 6 min after X-ray exposure. The 1:2:2:1 ratio of the asterisked four peaks (first, second, third, and fourth from the left) in Fig. 1 is a typical observation of DMPO-OH signals^33^. The outer two peaks indicate the signals of manganese oxide. The ESR signal heights of DMPO-OH were normalized with those of the Mn^2+^ signals.

**Figure 2:**
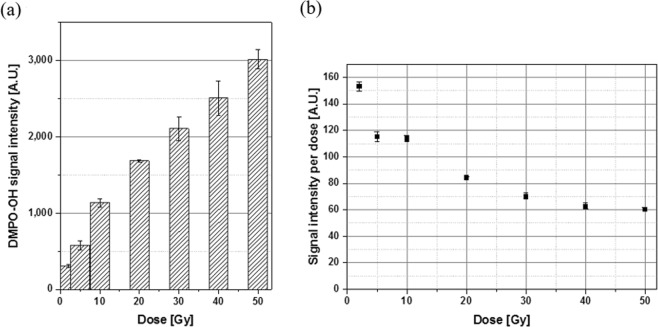
Average of the second and third peaks’ DMPO-OH signal intensities in an arbitrary unit measured at 6 min after 2–50 Gy of radiation exposures: (**a**) total intensity and (**b**) intensity per unit dose. Each error bar indicates one standard error of the mean obtained from three independent experiments.

The ESR signal intensity increases with increased DMPO-OH concentration^29–32^ and thus with increased radiation dose to the culture medium. We recorded the average of the second and third peak signal intensities while disregarding the small first and fourth peak values^34^. Figure 2(a) presents the average signal intensities observed after X-ray exposures at 2, 5, 10, 20, 30, 40, and 50 Gy. The signal intensity increased with X-ray dose despite the diminished efficiency of the unit dose with increased dose [Fig. 2(b)]. The smaller value of signal intensity per dose at a higher dose may be attributed partly to the relatively short half-life of DMPO-OH (14.5 min) as compared with the duration of experimental procedure. X-ray system delivered energy to the cells *in vitro* at a rate of 3.68 Gy/min and thus energy delivery upon exposures at 2 and 50 Gy took 1 and 13.6 min, respectively. An additional 6 min lapsed until ESR measurement after irradiation completion. Thus, approximately 7 min lapsed until ESR measurement after exposure to 2 Gy whereas approximately 19.6 min lapsed after exposure to 50 Gy. The total lapse from the start of DMPO-OH production by X-ray exposure until the ESR measurement increased with dose. The long lapse resulted in the dissociation of DMPO-OH products by a great portion. In consequence, the signal intensity per unit dose was low at a high dose.

### DMPO-OH production by APPJ treatment

The DMPO-OH signal production varies depending on the operating setup of the APPJ device and treatment duration^35^. In the APPJ system, electron density linearly increases with increased applied voltage^36^ and thus, more radicals such as OH are produced in plasma plume. Plasma species including those radicals are flown into the culture medium with gas flow^37^. Consequently, the OH radical concentration in culture medium increases.

**Figure 3:**
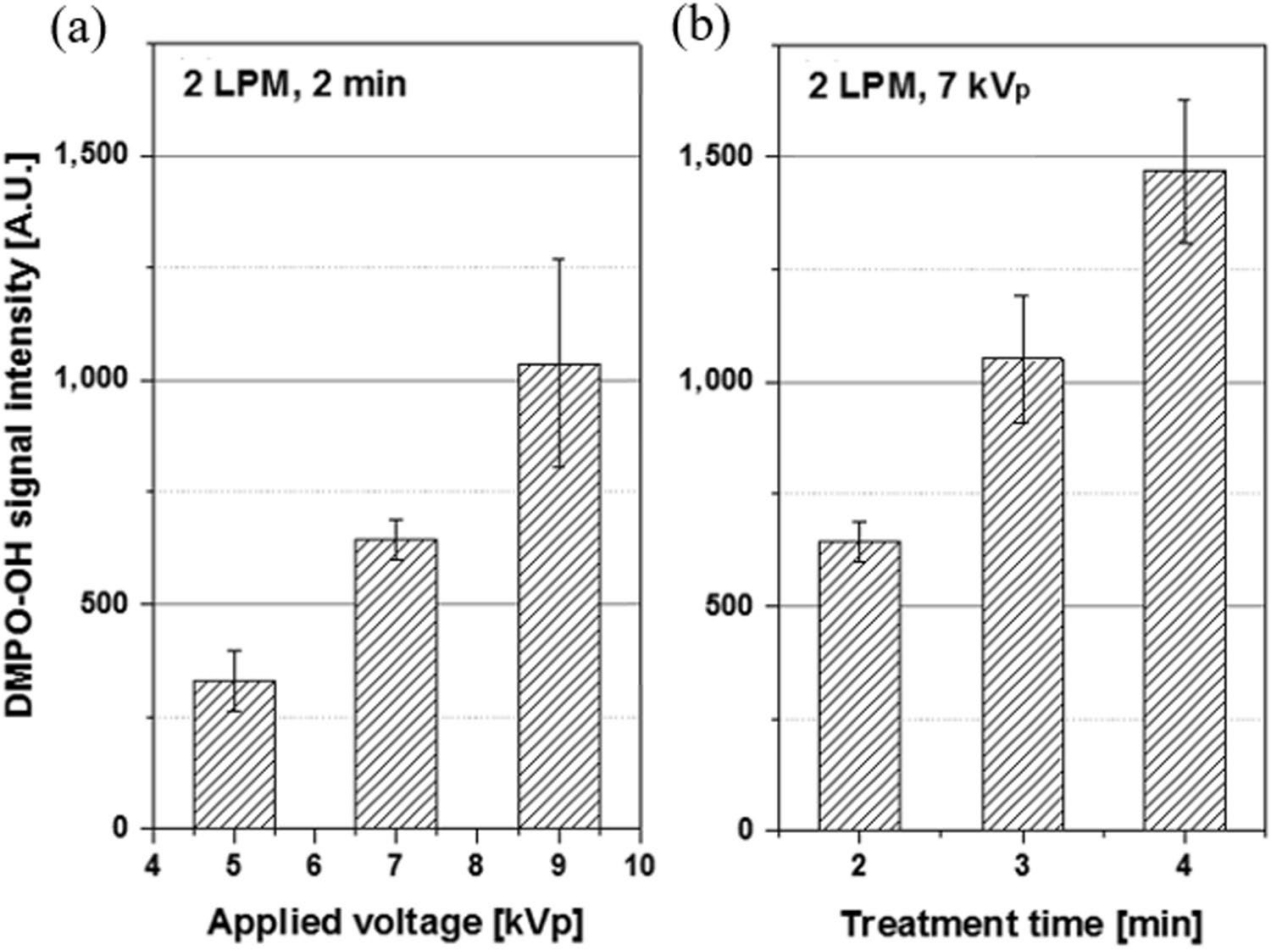
DMPO-OH signal intensities in an arbitrary unit: (**a**) from 2 min plasma treatments at the helium flow rate of 2 LPM with the applied voltage at 5, 7, and 9 kVp and **(b**) from 2, 3, and 4 min treatments at 2 LPM and 7 kVp. Each error bar indicates one standard error of the mean obtained from three independent experiments.

**Figure 4:**
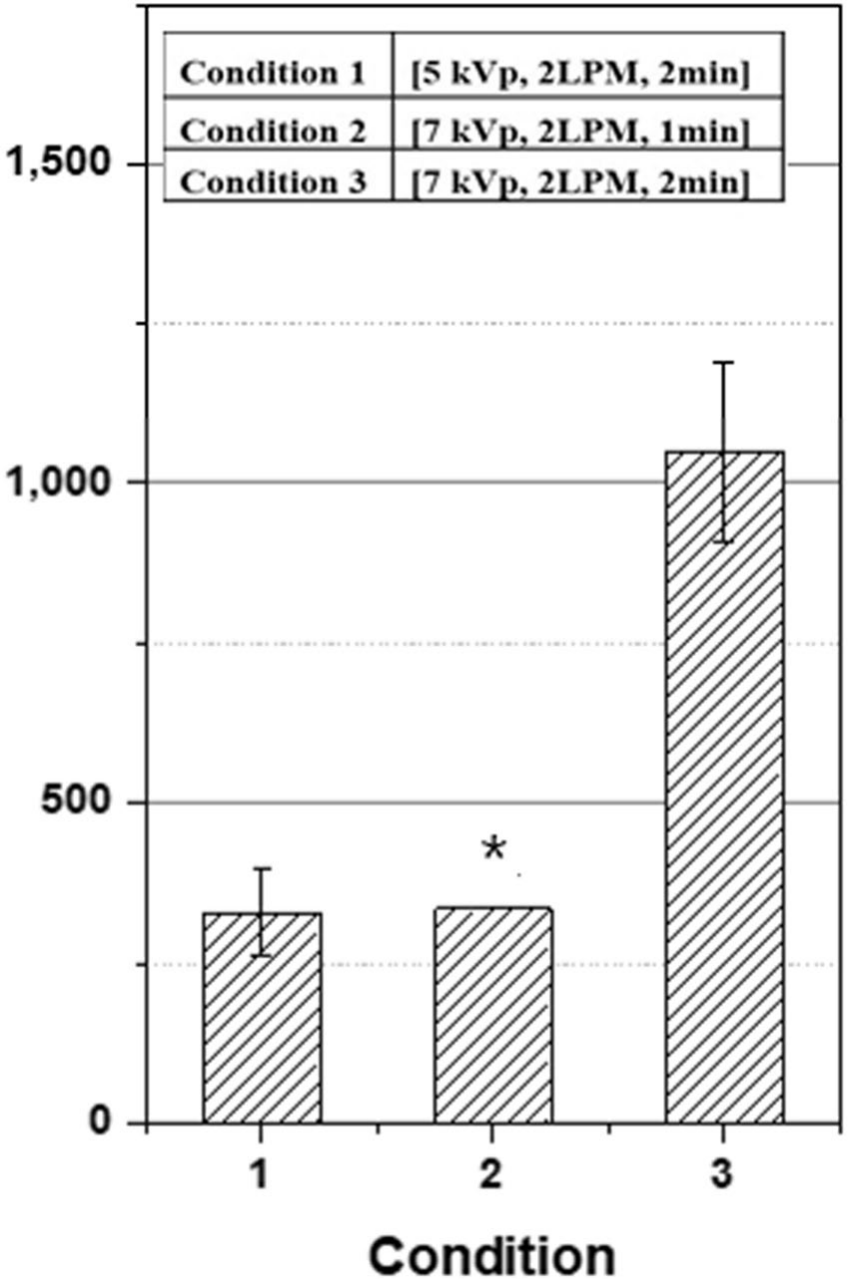
DMPO-OH signal intensities in an arbitrary unit from plasma treatments at three different operational setups of the plasma device in applied voltage, helium flow rate, and duration of treatment. The asterisked data for condition 2 was extrapolated from the data in Fig. 3(b). The data sets were normalized by external Mn^2+^ signal intensities. Each error bar indicates one standard error of the mean obtained from three independent experiments.

With the helium flow rate fixed at 2 liters per minute (LPM), the DMPO-OH signal intensity from 2 min of APPJ treatment increased proportionally with the applied voltage [Fig. 3(a)]. The signal intensity and the applied voltage showed a good correlation (R^2^ = 0.99). When the voltage and helium flow rate were fixed at 7 kVp and 2 LPM, respectively, the DMPO-OH signal intensity increased with prolonged duration of plasma treatment in a good correlation (R^2^ = 0.99) [Fig. 3(b)]. Plasma treatment lasted for 2, 3, and 4 min. Thus, the time lag including plasma treatment until ESR measurement ranged from 8 min to 10 min, which was shorter than the half-life (14.5 min) of the DMPO-OH signal. As a result, the DMPO-OH signal intensity was linearly proportional to the duration of plasma treatment. Figure 4 summarizes the DMPO-OH signal intensities under three operational setups of the APPJ device. The effect of voltage increase from 5 kVp to 7 kVp was offset by the effect of shortened (from 2 min to 1 min) treatment (condition 2 versus condition 1). The effect of voltage increase from 5 kVp to 7 kVp (condition 3 versus condition 1) was comparable with that of prolonged (from 1 min to 2 min) treatment (condition 3 versus condition 2).

### Intracellular ROS production by radiation and plasma treatments

The DMPO-OH signal intensity corresponds to the concentration of OH radicals in the cell culture medium. Notably, ROS molecules are cell-threatening when they are produced inside the cells^38^. Low-LET radiations, such as X-rays, are penetrating. Thus, the OH production by X-ray exposure would be uniform over the intra- and extra-cellular regions. However, plasma treatment leads to non-uniform OH radical production in cells and culture medium. The OH radical production by plasma treatment is explained in the following procedures^9,17,39^:

OH radicals produced in the gas flow near the medium surface are at very high concentrations. Highly reactive OH radicals are easily combined to form gaseous H_2_O_2_ radicals, which then dissolve into the medium. Otherwise, OH radicals are diffused into the medium and combined to form liquid H_2_O_2_. The H_2_O_2_ radicals of high stability can diffuse the medium deeply although OH radicals diffuse only a few μm^17^.

1 $$\cdot {\rm{OH}}+\cdot \,{\rm{OH}}\to {{\rm{H}}}_{2}{{\rm{O}}}_{2}$$

The production of OH radicals in the medium is mediated by the plasma-initiated UV emissions from the excited species near the medium surface. While penetrating the medium, UV dissociates H_2_O in the medium to form OH radicals directly or through excitation.

2 $${\rm{UV}}+{{\rm{H}}}_{2}{\rm{O}}\to \cdot \,{\rm{OH}}+\cdot \,{\rm{H}}$$

3 $${\rm{UV}}+{{\rm{H}}}_{2}{\rm{O}}\to {{\rm{H}}}_{2}{{\rm{O}}}^{\ast },\,{\rm{UV}}+{{\rm{H}}}_{2}{{\rm{O}}}^{\ast }\to \cdot \,{\rm{OH}}+\cdot \,{\rm{H}}$$

The relatively long-lasting and stable H_2_O_2_ radicals play the major oxidant in plasma treatment^14,39–43^. The OH radicals diffused from the gas flow into medium or produced near medium hardly reach the cells because of their short propagation range during the lifetime. Nevertheless, cold plasma has been reported to induce damage from organelles to DNA molecules^36–38^. A previous study has reported the mechanism that plasma treatment induces intracellular production of OH radicals via radical production outside the cells^33^. Proteins, such as ferritin and ferroportin, expressed in cells are able to catalyze H_2_O_2_ into OH radical by Fenton reactions.

4 $${{\rm{H}}}_{2}{{\rm{O}}}_{2}+{{{\rm{O}}}_{2}}^{-}\to {{\rm{OH}}}^{-}+\cdot \,{\rm{OH}}+{{\rm{O}}}_{2}$$

The abundant H_2_O_2_ radicals in the medium are transported into the cells through the membrane protein^44–50^, and superoxide radicals enter the cells by diffusion^51^. A recent study proved that aquaporin 8 expressed on the cancer cell membrane played as diffusion channels of non-thermal plasma-generated H_2_O_2_^52^. Several studies furthermore reported that extracellular H_2_O_2_ and O_2_^−^ were diffused into the cells and contributed to the increase of intracellular ROS in the treated cells^11,41,44^. The significance of intracellular ROS production by non-thermal plasma treatment has been confirmed through the observation that the cytotoxicity of non-thermal plasma was diminished with intracellular ROS scavangers^53–55^.

**Figure 5:**
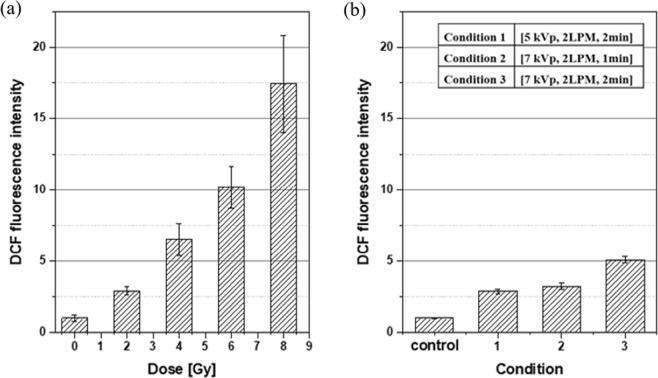
DCF fluorescence intensities varying (**a**) with the X-ray dose and (**b**) under three operating conditions of the APPJ device, both normalized to the control. Each error bar indicates one standard error of the mean obtained from three independent experiments.

The 2′,7′dichloro-dihydrofluorescein diacetate (H_2_DCF-DA) internalized to the cells are oxidized by ROS into 2′,7′dichlorofluorescein (DCF), which can be detected by flow cytometry. To differentiate the intracellular ROS concentration from the extracellular one, we incubated cells with H_2_DCF-DA in culture medium and measured the DCF fluorescence intensity produced exclusively inside the cells after each radiation exposure and plasma treatment. In mouse endothelial cells (MECs), DCF fluorescence intensity was observed to increase with increased X-ray dose in the range from 2 Gy to 8 Gy, as shown in Fig. 5(a). At 8 Gy of X-ray exposure, the DCF fluorescence intensity in the irradiated MECs was approximately 1750% of that in control cells. However, after plasma treatment, the DCF fluorescence intensity in MECs limitedly changed by enhancement of the applied voltage to plasma source device and extension of plasma treatment duration. As shown in Fig. 5(b), the DCF fluorescence intensity increased by less than twice by the voltage change from 5 kVp to 7 kVp (condition 3 vs. condition 1). Two times of plasma exposure duration (condition 3 vs. condition 2) resulted in approximately two times of DCF fluorescence intensity. This result is in contrast with the change to more than thrice in DMPO-OH signal intensity (Fig. 4).

### Comparison of radiation exposure and plasma treatment in ROS production

The OH radical production in culture medium was quantitated by DMPO-OH signal intensity (Figs. 2(a) and 4 for radiation and plasma treatments, respectively). Meanwhile, the intracellular production of ROS radicals was quantitated by DCF fluorescence intensity (Fig. 5(a,b) for radiation and plasma treatments, respectively). The DMPO-OH signal intensities were 329, 322, and 644 in an arbitrary unit (A.U.) under the APPJ operation at conditions 1, 2, and 3, respectively. The corresponding DCF fluorescence intensities were 300%, 344%, and 529% of the control, respectively.

**Figure 6:**
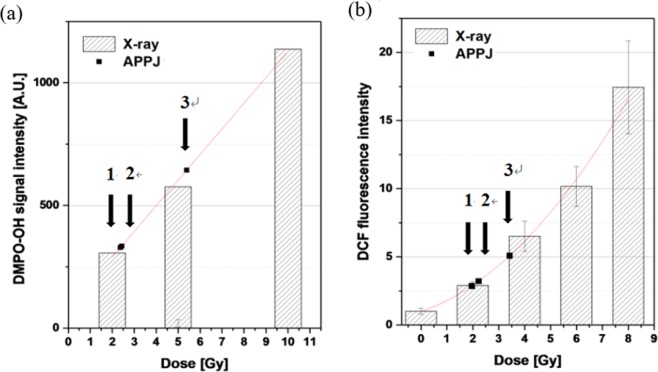
Effects of radiation exposure and plasma treatment in terms of (**a**) DMPO-OH signal intensity and (**b**) DCF fluorescence intensity. The DMPO-OH and DCF intensity data are fitted to a linear and a polynomial function, respectively. Arrows indicate the DMPO-OH and DCF intensities induced by plasma treatments under three operational setups (conditions 1, 2, and 3) of the APPJ device.

The plasma treatment conditions of 1, 2, and 3 were equivalent in OH radical production in the culture medium to the X-ray doses of approximately 2.34, 2.27, and 5.39 Gy, respectively, as indicated in Fig. 6(a). Regarding intracellular ROS production, the equivalent X-ray doses to the plasma treatment conditions 1, 2, and 3 were 1.96, 2.32, and 3.42 Gy, respectively [Fig. 6(b)]. Table 1 summarizes that the equivalent doses of conditions 1, 2, and 3 for the comparable DCF production (1.96, 2.32 and 3.42 Gy) are lower than for the comparable DMPO-OH production (2.34, 2.27 and 5.39 Gy), respectively. The lower equivalent doses of plasma treatment for DCF production imply that plasma treatment is less efficient in intracellular ROS production as compared for OH production in the culture medium. Notably, intracellular ROS radical production is attributed to direct energy delivery of radiation to cellular molecules, whereas it is attributed to indirect plasma action via H_2_O_2_ production in the culture medium (Eq. (4)).

**Table 1:** DMPO-OH signal and DCF fluorescence intensities under three different parametric setups of APPJ device operation and the X-ray doses that would cause the comparable DMPO-OH and DCF signal intensities to plasma treatments.

Operational conditions^*^ of APPJ device		DMPO-OH signal intensity (AU)	DCF fluorescence intensity (% of the control)	X-ray dose^†^ for the comparable DMPO-OH production	X-ray dose^‡^ for the comparable DCF production
**1**	(5 kVp, 2 LPM, 2 min)	329 ± 66^**^	287 ± 15	2.37 Gy	1.96 Gy
**2**	(7 kVp, 2 LPM, 1 min)	335 ± 13^※^	321 ± 22	2.43 Gy	2.22 Gy
**3**	(7 kVp, 2 LPM, 2 min)	644 ± 43	509 ± 20	5.39 Gy	3.42 Gy

^*^(applied voltage, helium gas flow rate, duration of treatment).

^**^standard error.

^†^X-ray dose derived as in Fig. 6(a).

^‡^X-ray dose as in Fig. 6(b).

^※^Data obtained by extrapolation from the data in Fig. 3(b).

### Comparison of clonogenic cell deaths from X-ray exposure and plasma treatment

**Figure 7:**
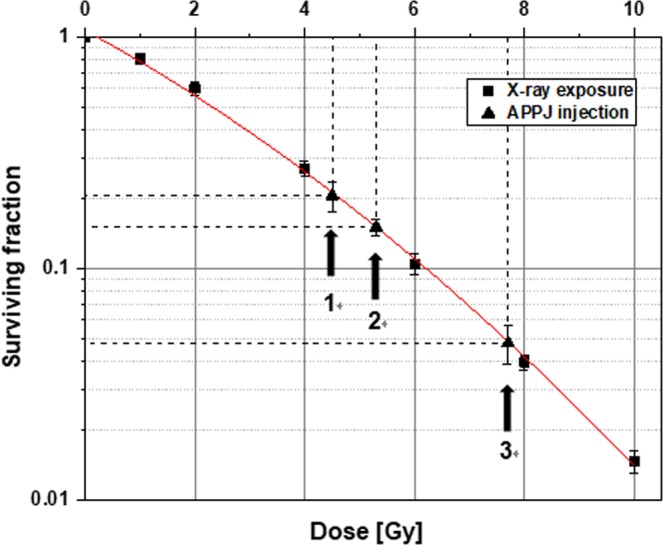
Surviving fractions of MECs from X-ray exposures of up to 10 Gy and plasma treatments under operational conditions 1, 2 and 3 of the APPJ device. The experimental data (solid squares) from radiation exposure were fitted to the solid curve. The solid triangles mark the clonogenic surviving fractions of MECs from plasma treatments under three different conditions 1, 2 and 3 of the APPJ device operation. Each error bar indicates one standard error of the mean obtained from three independent experiments.

The clonogenic surviving fractions of MECs from X-ray exposure at doses of up to 10 Gy are presented in Fig. 7. The experimental data (solid squares in Fig. 7) were fitted to a linear-quadratic curve (solid line in Fig. 7): surviving fraction (SF) = exp [–0.223*D*–0.023*D*^2^]. The surviving fractions of cells from plasma treatments under conditions 1, 2, and 3 were marked (solid triangles in Fig. 7) on the fitting curve. Radiation doses corresponding to those SF values under conditions 1, 2, and 3 were read at 4.5, 5.3, and 7.3 Gy, respectively (see Table 2).

**Table 2:** Surviving fractions of MECs from plasma treatments under three different operational conditions of the APPJ device and the approximate X-ray doses that would cause the comparable fractions of clonogenic cell death.

Operational conditions^*^ of APPJ device		Clonogenic surviving fraction (SF)	Fraction of clonogenic cell death (1–SF)	X-ray dose for comparable fraction of clonogenic cell death (1–SF)
**1**	(5 kVp, 2 LPM, 2 min)	0.206 ± 0.029^**^	0.794	4.5 Gy
**2**	(7 kVp, 2 LPM, 1 min)	0.151 ± 0.013	0.849	5.3 Gy
**3**	(7 kVp, 2 LPM, 2 min)	0.048 ± 0.009	0.952	7.7 Gy

^*^(applied voltage, helium gas flow rate, duration of treatment).

^**^standard error.

### Equivalent dose of X-ray exposure to plasma treatment in cellular effect

**Figure 8:**
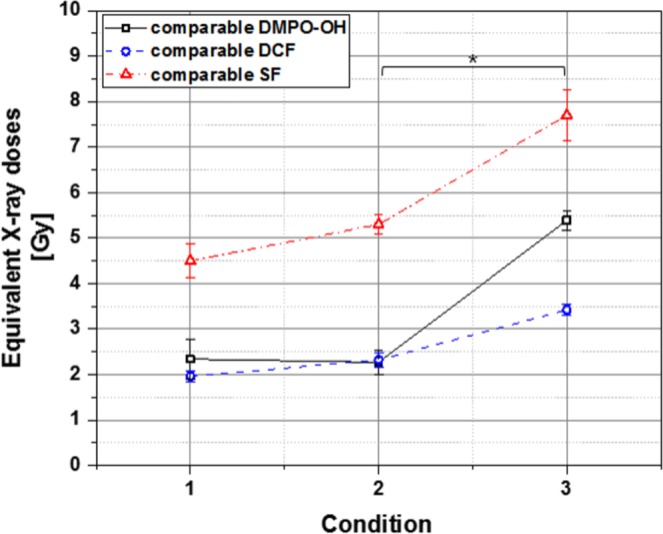
The *equivalent X-ray doses* in DCF fluorescence and DMPO-OH signal productions, and the fractional clonogenic cell death observed in MECs after plasma treatment under three (1, 2, and 3) operational conditions of APPJ device. Each data point was obtained from three to five independent experiments. Error bars indicate one standard error for individual mean values. The asterisk indicates that the equivalent X-ray doses of operational conditions 2 and 3 are significantly different from each other (p < 0.05) with regard to medium OH and intracellular ROS productions and clonogenic cell death.

Figure 8 summarizes the radiation doses that would result in the equivalent OH radical production in the cell culture medium, intracellular ROS production or clonogenic cell death to our observations after plasma treatments under three different conditions. The *equivalent X-ray dose* indicates the level of X-ray exposure that would result in the comparable amount of intracellular ROS or medium OH production, or the comparable fraction of clonogenic cell death (=1–SF) to the plasma treatment of different conditions in the APPJ device operation. Considering that non-thermal plasma and X-rays are common in point of inducing cell death through ROS (especially OH radical) production^1,6,9,10^, a high fraction of clonogenic cell death can be expected under the plasma condition that causes a great amount of ROS or OH production. Overall, our observations in Fig. 8 comply with the expectation. Plasma treatment under condition 3, among three conditions, corresponds to the highest values of equivalent X-ray dose in medium OH and intracellular ROS production and in the fraction of clonogenic cell death.

Figure 8 shows that each of plasma treatment conditions 1, 2, and 3 corresponds to a higher value of equivalent X-ray dose in causing clonogenic cell death than in producing medium OH or intracellular ROS. This result means that plasma treatment was more efficient in causing clonogenic cell death per unit production of medium OH or intracellular ROS than X-ray exposure.

In plasma treatment under condition 2, the equivalent X-ray dose in DMPO-OH signal production (2.43 Gy) is comparable with that in DCF fluorescence generation (2.22 Gy). This finding means that plasma treatment under condition 2 produced DMPO-OH signal and DCF fluorescence at similar intensity ratios to the DMPO-OH signal and DCF fluorescence, respectively, induced by X-ray exposure. In plasma treatment under condition 3, the equivalent X-ray dose in DMPO-OH signal production (5.39 Gy) is higher than that in DCF fluorescence generation (3.42 Gy). This finding shows that the increased duration from 1 min to 2 min of plasma treatment was effective for increasing the production more of medium OH than of intracellular ROS. The *equivalent radiation dose* in DMPO-OH signal production increased by over 120%, whereas that in DCF fluorescence generation increased by approximately 55% (Table 1). Notably, the equivalent dose with regard to the fraction of clonogenic cell death increased by approximately 45% (5.3 Gy to 7.7 Gy) due to the increased duration of plasma treatment from 1 min to 2 min (Table 2). Clonogenic cell death is attributed presumably to the intracellular ROS production rather than to the medium OH production.

In plasma treatment condition 2 as compared with the condition 1, the voltage applied to the APPJ device was raised from 5 kVp to 7 kVp, whereas the treatment duration was reduced from 2 min to 1 min. The DMPO-OH signal (and thus its equivalent radiation dose) changed by less than 2%, whereas the DCF fluorescence intensity increased by approximately 13% (Table 1). Clonogenic cell death increased by approximately 7%. The loss in intracellular ROS production caused by reduced treatment duration was compensated by the great effectiveness of intracellular ROS production at a high voltage. The high voltage compensated the reduction of medium OH production caused by reduced treatment duration to a less extent. Overall, the net increase in intracellular ROS production resulted in the increased clonogenic cell death. A previous study informed that apoptotic cell death was switched to necrotic cell death with increased applied voltage^56^.

## Conclusion

Medium OH and intracellular ROS productions were considered as common indices for the bioeffects of radiation and plasma. The *equivalent X-ray dose* to each operational setup of APPJ device was defined as the X-ray dose that would induce comparable radical production or the comparable fraction of clonogenic cell death to plasma treatment. The operational setup of the APPJ device inducing a great radical (medium OH or intracellular ROS) production corresponded to the high fraction of clonogenic cell death. In plasma treatment, the clonogenic cell death was better predicted by intracellular ROS production rather than by medium OH production.

Considering that different cell lines showed different radiosensitivity to X-ray exposures^57,58^, we presume that equivalent radiation doses of other cell lines to the same plasma treatment might be different. Nevertheless, the mechanism of inducing clonogenic cell death via intracellular ROS production would still apply to other cell lines.

## Methods

### Cell line and cell culture

MECs (CRL-2161, ATCC, Manassas, VA, USA) were cultured in T-25 flask (Nunc, Roskilde, Denmark) containing Dulbecco’s modified eagle medium (DMEM) (Gibco, Grand Island, NY, USA) supplemented with 10% (v/v) heat-inactivated fetal bovine serum (FBS) (Gibco). Cells were incubated at 37 °C in a humidified incubator (MCO-230ALC, Panasonic, Gunma, Japan) with 10% CO_2_. Before exposure to X-ray or plasma, cells were washed twice with phosphate-buffered saline (Invitrogen, Carlsbad, CA, USA) and prepared into single-cell suspensions with TrypLE Express (Gibco).

### Radiation exposure and plasma treatment

**Figure 9:**
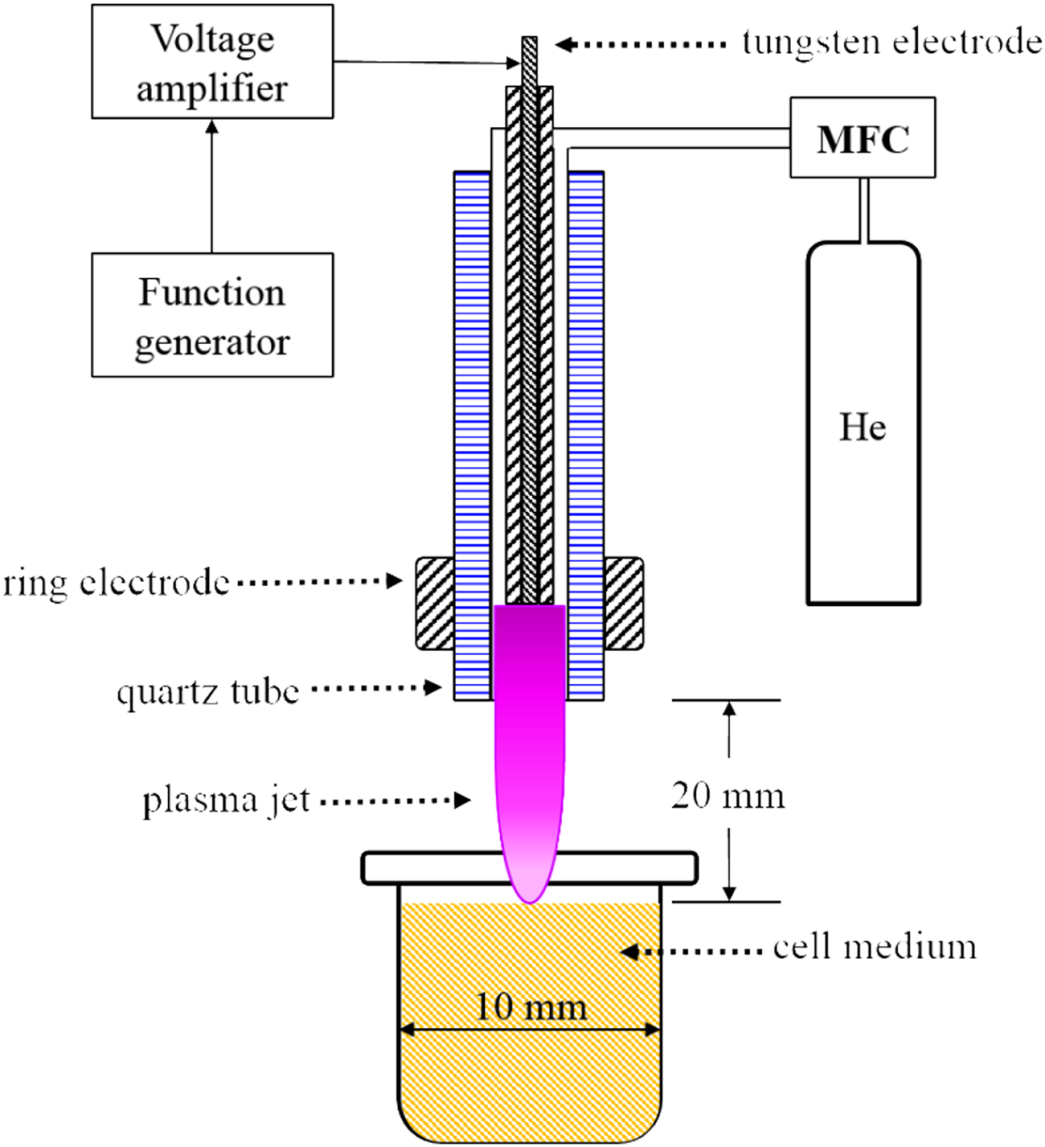
A schematic of APPJ injection into cell culture medium. The helium gas-flow rate is controlled by a mass flow controller. The high electric field produced between two electrodes induces breakdown of the helium gas. Then, the plasma propagates along the tube over the cell culture medium.

Cells in the culture medium were exposed to X-rays at 2–50 Gy by operating the X-ray tube (450-D08, YXLON, Hamburg, Germany) at 350 kVp and 10 mA. Dose rate was 3.68 Gy/min. The non-thermal plasma injection was made by operating the APPJ device. Plasma production was controlled by varying the voltage applied to the pin electrode with a function generator (33220A, Agilent, Santa Clara, CA, USA) at 20 kHz and a voltage amplifier (20/20C, Trek, New York, NY, USA) at 2000 times amplification The applied voltage was changed from 5 kVp to 7 kVp and to 9 kVp. The helium gas-flow rate was fixed at 2 LPM using a mass flow controller (TN2911V-4S, Celerity, Allen, Texas, USA). The 10 mm-diameter TubeOne^®^ microcentrifuge tubes (S1620-2700, STARLAB, Hamburg, Germany) were filled with 2.0 ml culture medium containing 2 × 10^5^ cells. The distance between the nozzle of APPJ device and the medium surface was fixed at 20 mm. The plasma fluence over the cells and culture medium was further controlled by varying the duration of plasma treatment. Figure 9 depicts the schematic of the APPJ injection into cell culture medium.

### Measurement of DMPO-OH signal intensity

We used an ESR spectrometer (JES-TE200, JEOL, Tokyo, Japan) to detect DMPO-OH signals from the culture medium treated with X-rays or plasma. DMPO (D5766, Sigma Aldrich, St. Louis, MO, USA) was dissolved in DMEM (Gibco) supplemented with 10% (v/v) heat-inactivated FBS (Gibco), which was then added to the culture medium. The medium containing 100 mM DMPO was placed in the 2.0 ml TubeOne^®^ microcentrifuge tubes (S1620-2700, STARLAB, Hamburg, Germany) and exposed to either X-rays or plasma. The samples were analyzed as quickly as allowable after treatment. The ESR spectrometer was operated at 341.0 mT ± 10 mT (magnetic field), 1 mW (power), 9.42 GHz (frequency), 200-fold (amplitude), and 2 min of sweep time. A manganese signal was used for standardizing the external signal.

### Detection of intracellular ROS

Cells were incubated with 50 μM H_2_DCF-DA (ab113851, Abcam, Cambridge, UK) in culture medium for 30 min at 37 °C and 10% CO_2_. The H_2_DCF-DA-loaded cells were exposed to either X-rays or plasma and then incubated for 30 min. The operational setups in applied voltage, helium gas-flow rate, and treatment time of the plasma device were 5 kVp, 2 LPM, and 2 min in condition 1, 7 kVp, 2 LPM, and 1 min in condition 2, and 7 kVp, 2 LPM, and 2 min in condition 3. Cells were analyzed using a flow cytometry system (FACS Aria^TM^, BD Biosciences, San Jose, CA, USA) to measure the fluorescence.

### Clonogenic assay

Cellular responses to radiation and plasma were determined by clonogenic assay^59^. After exposure to radiation or plasma, cells were plated onto 35 mm culture dishes (Nunc) containing 3 ml of culture medium and incubated for 12 days. During incubation, the medium was replaced with a fresh one every 3 days. At the end of 12-day incubation, the cells were fixed with 70% ethanol and stained with 5% Giemsa solution (Sigma Aldrich). Colonies were counted with naked eyes. The colonies of more than 50 cells were taken as clonogenic survivors.

When cells are seeded as a single cell suspension in culture medium at low densities, they may grow into colonies. The percentage of colony-forming cells among the seed single cells is the plating efficiency (PE in Eq. (5)). The clonogenic surviving fraction (SF in Eq. (6)) was calculated by dividing the PE after X-ray or plasma treatment by the PE of the control cells.

5 $${\rm{PE}}=\frac{{\rm{Number}}\,{\rm{of}}\,{\rm{colonies}}\,{\rm{counted}}}{{\rm{Number}}\,{\rm{of}}\,{\rm{cells}}\,{\rm{seeded}}}\times 100\,( \% )$$

6 $${\rm{SF}}=\frac{{\rm{Number}}\,{\rm{of}}\,{\rm{colonies}}\,{\rm{counted}}\,{\rm{after}}\,{\rm{tretment}}}{{\rm{Number}}\,{\rm{of}}\,{\rm{cells}}\,{\rm{seeded}}\times {\rm{PE}}}\times 100\,( \% )$$

### Statistical analysis

Student’s t-test was performed to evaluate the significance of the difference between observed data. Data were judged to be significantly different from each other when p-value was less than 0.05.
